# Protocol for measuring the effects of an inhibitory signal associated with danger on honey bee dopamine levels

**DOI:** 10.1016/j.xpro.2024.103230

**Published:** 2024-08-01

**Authors:** Shihao Dong, Gaoying Gu, Tao Lin, Ziqi Wang, Jianjun Li, Ken Tan, James C. Nieh

**Affiliations:** 1 CAS Key Laboratory of Tropical Forest Ecology, Xishuangbanna Tropical Botanical Garden, Chinese Academy of Sciences, Kunming, Yunnan 650000 China; 2 University of Chinese Academy of Sciences, Beijing 100049, China; 3 Division of Biological Sciences, Section of Ecology, Behavior, and Evolution, University of California San Diego, La Jolla, CA 92093, USA

**Keywords:** Environmental sciences, Evolutionary biology, Model Organisms

## Abstract

The stop signal is produced in response to negative experiences at the food source and inhibits honey bee (*Apis mellifera*) waggle dancing. Here, we present a protocol for measuring the effects of an inhibitory signal associated with danger on honey bee dopamine levels. We describe steps for observing honey bee colonies, training them with artificial nectar, and simulating hornet attacks. We then detail procedures for recording waggle dancing and stop signals and measuring brain dopamine levels during different treatments.

For complete details on the use and execution of this protocol, please refer to Dong et al.^1^

## Highlights

• Setting up observation colonies and training foragers to a feeder
• Steps for measuring forager responses before and after hornet attacks
• Procedure for quantifying bee brain dopamine levels
• Increasing brain dopamine levels and studying the effect on fear-like behavior

## Abstract (editor highlights)

Publisher’s note: Undertaking any experimental protocol requires adherence to local institutional guidelines for laboratory safety and ethics.

## Graphical abstract

**Figure:**
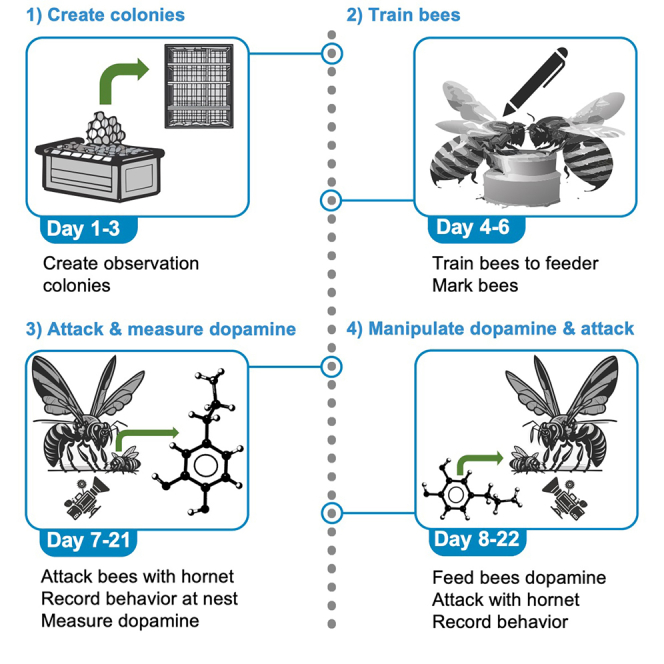

## Teaser

The stop signal is produced in response to negative experiences at the food source and inhibits honeybee (*Apis mellifera*) waggle dancing. Here, we present a protocol for measuring the effects of an inhibitory signal associated with danger on honeybee dopamine levels. We describe steps for observing honeybee colonies, training them with artificial nectar, and simulating hornet attacks. We then detail procedures for recording waggle dancing and stop signals and measuring brain dopamine levels following different treatments.

## Before you begin

This protocol outlines the steps required to investigate the impact of predation and dopamine supplementation on dopamine levels in the heads of honey bees and their signaling behaviors, using honey bee colonies.

1. Begin by securing multiple healthy *Apis mellifera* honey bee source colonies from a beekeeper.

***Note:*** Each colony should consist of at least 20 Langstroth comb frames (each frame is 23.18 cm high, 3.49 cm wide, and 48.26 cm long) housed inside two standard wood box bodies (each box is 25.4 cm high, 53.34 cm wide, and 41.59 cm deep) and containing approximately 45,000 bees.

***Note:*** Ensure they are abundantly supplied with food, pollen, and brood.

***Note:*** These will form the basis of your observation colonies.

2. Choose an appropriate time of year when foragers are active and freely foraging outside.

**CRITICAL:** It is crucial to avoid adverse weather conditions like cold or rain that prevent bees from being trained at feeders. Conversely, periods of high floral abundance may make training to artificial feeders difficult. Opt for times of moderate floral availability and good weather, typically early fall or its equivalent in your region. Please see detailed instructions and troubleshooting suggestions below.

3. Verify the availability of predators intended for use in your experiments within the chosen experimental time frame. The absence of hornets, for example, during your planned field season would preclude conducting these experiments.
4. Prepare by collecting all necessary equipment and materials as outlined in subsequent sections of this protocol.

### Creating honey bee observation colonies

**Timing: 1 day per colony (3 days for 3 colonies)**

#### Preparation of observation hives

**Figure 1:**
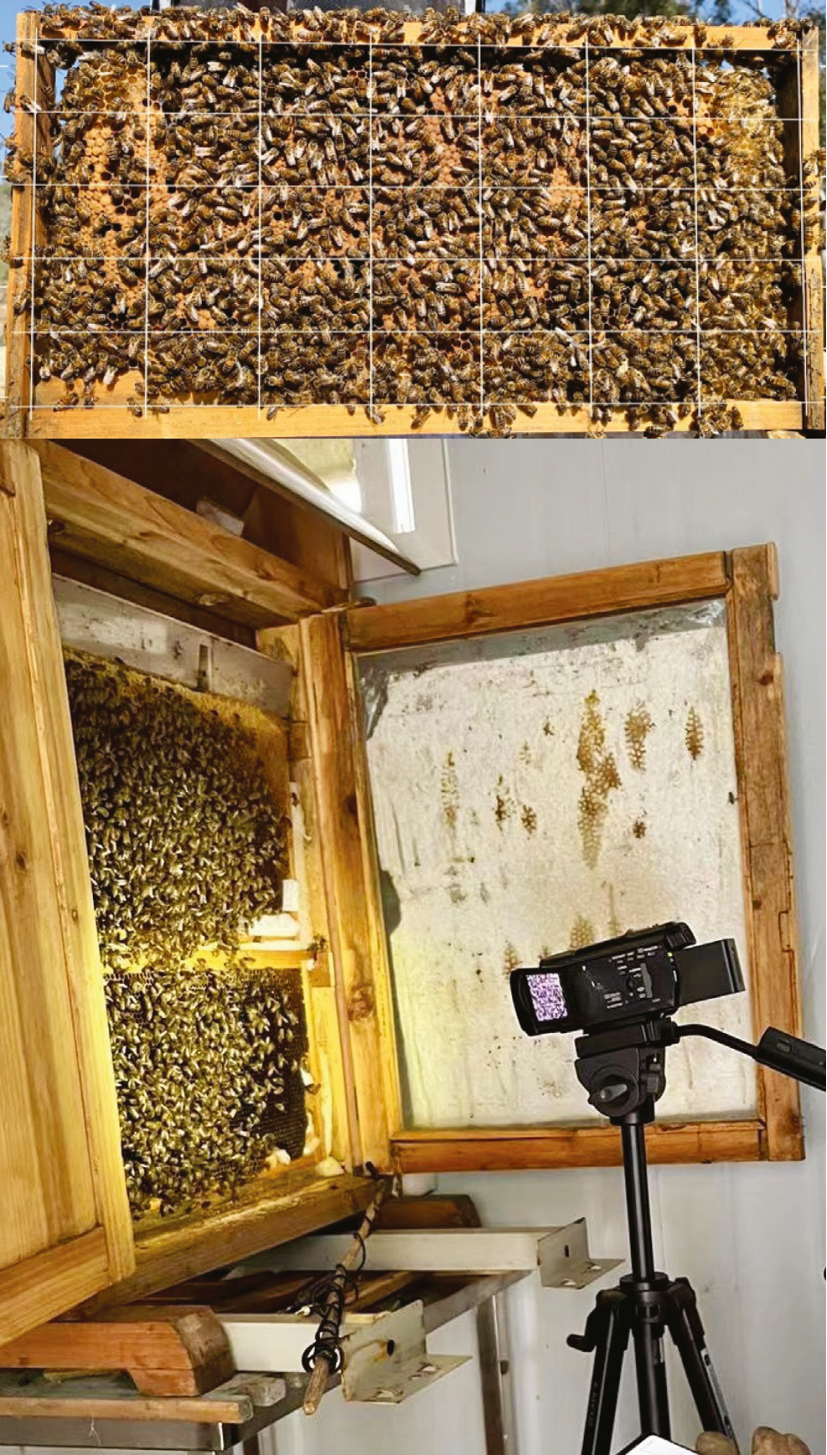
A good comb with a large number of bees and brood for setting up an observation colony (above) and a typical two-frame honey bee observation colony with the setup to record bee behaviors (below) are shown. A video camera allows the observer to record bee behaviors, and a wood rod holding the microphone is shown resting near the base of the colony.
  b. Ensure each comb includes food, brood, and adult bees from the original colony, with one frame dedicated to brood (80% coverage) and the other to honey (50% coverage) and pollen (50% coverage).
6. Install a tube with an inner diameter of 2–3 cm and a length of 25 cm through the wall to connect the observation hive to the exterior, facilitating bee entry and exit.
7. Insert a divider made of wood and beeswax within the hive to guide incoming bees toward one side of the lower comb.

***Note:*** Nieh^2^ provides a detailed schematic of such a design. This designated area acts as the dance floor for the bees to perform waggle dances, enabling observation of their communication behaviors.^2^

5. Construct each observation hive with dimensions of 55.4 cm in length, 17 cm in width, and 64 cm in height.
  a. Incorporate two combs measuring 43.5 cm by 23 cm from a fully established honey bee colony, as illustrated in Figure 1.

#### Preparation of colonies

8. Create each observation hive by selecting two combs from a complete honey bee colony. Make sure these combs include food, brood, adult bees, and the queen from the original colony to ensure a comprehensive inclusion of worker castes. Figure 1 shows such a comb.
9. Access the interior of the observation colony by carefully removing the glass cover and any other protective elements such as doors, as illustrated in Figure 1**.**
10. With care, insert the combs into the observation colony, verifying their stability and correct alignment within their new environment.
11. Execute the aforementioned steps (8–10) to establish a total of three observation colonies, which will serve as the basis for the research study.

**CRITICAL:** After the colonies are established, give them at least three days of undisturbed rest before using them.

### Training foragers to a feeder

**Timing: about 1 day per colony (3 days for 3 colonies)**

**Figure 2:**
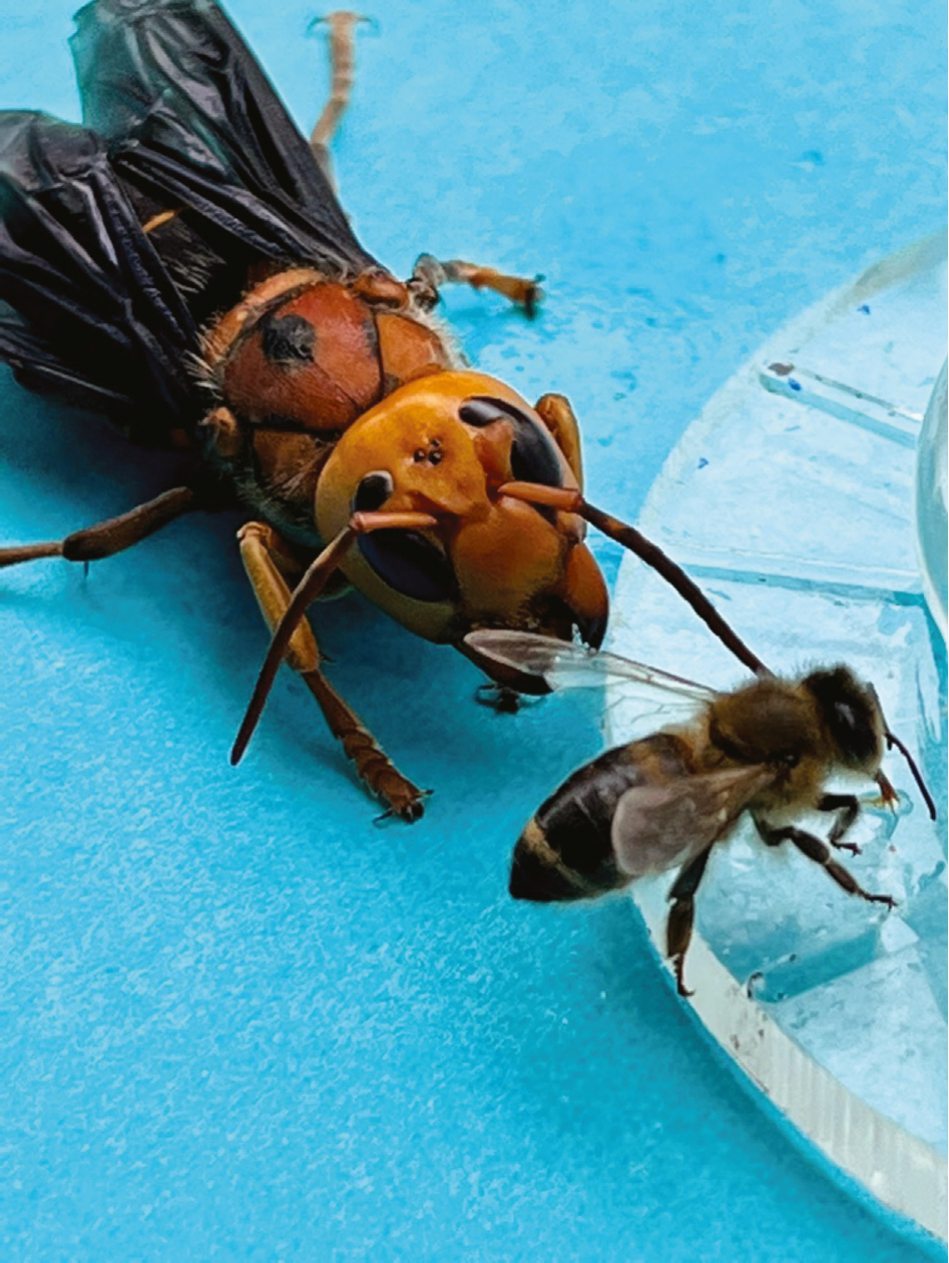
The standard grooved-plate sucrose solution feeder used to feed honey bee foragers. In this experiment, the forager has been feeding on sucrose solution with dopamine hydrochloride for 30 min and now has significantly elevated brain dopamine levels. This photo demonstrates that even a tethered *Vespa mandarinia* hornet, from which the forager would normally flee, does not elicit alarm behavior. The bee continued to feed.

***Note:*** Most researchers design and fabricate their feeders. However, you can download a 3D design of such a feeder base (https://cults3d.com/en/3d-model/tool/grooved-plate-classic-bee-feeder-for-research) and print it yourself or have it printed at a 3D printing facility. A transparent disk is most versatile since one can change the color by placing a sheet of colored circular plastic underneath the disk.

12. The necessary time for training varies based on the colony’s state and foragers' access to natural floral resources.
  a. When natural floral resources are scarce, researchers can train bees to a feeder at a distance of 120 m from the focal colony within a few hours.
    ***Note:*** The waggle dance is a behavior that spans round dancing (which does not communicate food location) when food is nearby to a waggle dance which communicates food location. This transition is typically complete after approximately 100 m is reached,^3^ and thus we suggest 120 m to ensure that foragers are performing clear waggle dances.
  b. When floral resources are abundant, training may span several days.
  c. Train bees from each colony to a different location to help ensure that bees at a given feeder location are from the focal colony being used at that time.
  d. Train with only one colony at a time. This is the focal colony.
13. First, set up the feeder using a 60 mL vial, 5.2 cm in height (Fisher Scientific catalog number 02-911-773), flipped over a custom-made circular transparent plastic disk (10 cm diameter) equipped with 18 feeding grooves (1 mm deep, 1 mm wide, and 50 mm long) to allow sucrose solution to flow through (as shown in Figure 2).

14. Fill the vial with a 2.5 M sucrose solution, invert it onto the plastic disk, and position it atop a yellow paper circle (10 cm diameter) to ensure the feeder can host more than 30 foragers without causing congestion.
15. As bees exit the nest, use a vial to capture them gently (described in detail below), identifying them as likely foragers.
  a. Transport the captured bee to a feeder located 120 m from the colony.
  b. Gently release the bee by placing the open side of the vial down on the disk of the grooved plate feeder.
  c. Slowly lift the vial as the bee starts to drink from the feeder.
  d. Once the bee begins feeding, apply a distinctive color mark on its dorsal thorax using a paint pen (such as Edding 750).

***Note:*** You can mark each bee with multiple colors at different positions to create unique, individual marks.

16. Allow the marked bee to return to the nest.
  a. If the bee does not come back to the feeder, continue to capture and mark other bees using the same method until at least 15 bees consistently return to the feeder within 20 min.
  b. At this point, you can begin the experiment. Work with only one colony at a time.

## Key resources table

**Table:** 

REAGENT or RESOURCE	SOURCE	IDENTIFIER
**Chemicals, peptides, and recombinant proteins**		

Sodium 1-octanesulfonate	Macklin	S817852-25g
EDTA	BioFroxx	1340GR500g
Citric acid	Chemical Reagent	500g
NaH_2_PO_4_	Macklin	S817780-500g
Acetonitrile	Mreda	M157-4L
ddWater		
Na_2_S_2_O_5_	Macklin	S818096-100g
HClO_4_	Chemical Reagent	500mL
Dopamine hydrochloride	J&K	62-31-7

**Other**		

HD (1280 × 720 pixels) Video camera	Sony	Model HDR-PJ790
Honey bee (*Apis mellifera ligustica*) colony with a queen and female and male bees (all ages)	Bee breeder	*Apis* *mellifera* *ligustica* Spinola, 1806
Movo LV1 lavalier microphone	Amazon.com	ASIN B00HX40Q3C
Electric grinder	Tiangen	OSE-Y50
Centrifuge	Eppendorf	5424R
HPLC-ECD system	Waters	1525 binary HPLC pump
	Waters	Autosampler Waters 2707
	Waters	C 18 reversed-phase column
	Waters	UV detector UV/visible detector, Waters 2489
	Scientz	Ultrasonic instrument SB-800D

## Materials and equipment

**Table:** Protein precipitation solution

Reagent	Final concentration	Amount
Perchloric acid (0.4 mol/L)	2.4 mL /100 mL	2.4 mL
Na_2_S_2_O_5_ (2.6 mmol/L)	0.0494 g/100 mL	0.0494 g
EDTA (2.7 mmol/L)	0.0789 g/100 mL	0.0789 g
ddH_2_O	N/A	add to 100 mL
**Total**	**N/A**	**100 mL**

Store at –4°C for up to 3 months.

**Table:** Mobile phase

Reagent	Final concentration	Amount
Acetonitrile (100 mL/L)	100 mL/L	100 mL
Sodium 1-octane sulfonate (1.7 mmol/L)	1.7 mmol/L	0.3677 g
Anhydrous sodium dihydrogen phosphate (64 mmol/L)	64 mmol/L	7.6787 g
EDTA (50 μmol )	50 μmol/L	0.0146 g
Citric acid	N/A	N/A
pH (3.0)	N/A	adjust with citric acid
ddH_2_O	N/A	to 1 L
**Total**	**N/A**	**1 L**

Prepare and use immediately. Do not store.

**CRITICAL:** Perchloric acid can cause a fire or explosion and is a strong oxidizer. It may be corrosive to metals and is harmful if swallowed. It causes severe skin burns and eye damage. Keep away from heat, hot surfaces, sparks, open flames and other ignition sources. No smoking is allowed. Wear protective gloves, protective clothing, eye protection, and face protection.

## Step-by-step method details

### Effect of predator threat on waggle dance and stop signal

**Timing: 14 days (approximately 7 h per day)**

1. Begin training 30 bees from each colony to approach feeders filled with a 50% sucrose solution (w/v), situated 120 m away from their respective colonies.
  a. At the hive entrance, carefully capture bees that are leaving in a vial, and gently release them at the feeder by turning the open vial over the feeder.
  b. Keep the vial in place until the bee starts to feed.
  c. To minimize competition, train bees from different colonies in separate directions towards distinct feeders.
2. While the bees are feeding, mark each one on the thorax with different colors using paint pens (such as Edding 750) to ensure they can be identified as belonging to the specific colony.
3. Employ an aspirator to gently capture bees from the feeding area as necessary to prevent overcrowding.

***Note:*** You will retain bees in the aspirator until the end of the day and then release them. However, if you find that a large number of bees return the next training day, it will be necessary to freeze the bees in the aspirator and dispose of them.

4. Have a second researcher monitor the bees as they return to the observation colony.

***Note:*** A bee is trained when it returns on its own to the feeder. After several trips to the feeder, foragers are expected to initiate waggle dances to communicate the feeder's location to nestmates.

***Note:*** Karl von Frisch devised an excellent system in which colors and their orientation on the dorsal thorax correspond to numbers.^4^ This system can be quite useful for individually identifying a large number of bees.

***Note:*** Have a paper towel handy to blot the paint pen tip periodically to ensure a controlled, small, clear mark. The same towel blotting technique is useful if you opt to use paint brushes instead of paint pens. For very fine marks, pieces of straw or grass dipped into the paint on the tip of the pen can also be used.

***Note:*** To control the number of bees at a feeder, have a clipboard with a sheet of paper upon which the observer writes the identity of the bees in separate columns and then, if they arrive within a 15-min interval, as rows. This censusing method is a classic design of Tom Seeley.^5^

**CRITICAL:** Training time will depend upon floral resource conditions and the weather. Be patient and make sure you do not disturb bees when capturing them or releasing them.

5. After bees have been trained to the feeder, simulate hornet attacks without causing harm to foragers through the following method:
  a. Initially, capture a hornet (*Vespa mandarinia*) using an insect net during its attacks on other colonies within the apiary.
  b. To keep the hornet in optimal condition for several days, house it in a cage with access to a 50% (v/v) sucrose solution at room temperature.
  c. Secure the hornet (*V. mandarinia*) to the end of a 50 cm long and 5 mm diameter wood rod using a stiff wire, wrapping it between the hornet’s thorax and abdomen as demonstrated in Methods video S1**.**
    Methods video S1. How to tether hornets, related to step 5 This video demonstrates how to tether a *V. mandarinia* hornet that has been previously captured and kept in a box. Please note that this video records its tethering by an extremely experienced and proficient researcher who has spent over a decade working with these hornets. We recommend wearing full hornet protective gear and hornet-proof gloves when attempting this procedure and also working, as shown here, near a large bank of open windows through which a hornet can easily fly out if it escapes the tweezers.
  d. This setup permits the hornet to flap its wings, mimicking the motion observed during natural attacks.
  e. Position the tethered hornet approximately 2 cm away from the feeder, ensuring it does not come into contact with the bees (refer to Figure 2).
    ***Note:*** At this distance, bees typically exhibit alarm behaviors and tend to flee.
  f. Instruct the person monitoring the feeder to gently move the rod at a speed of about 0.2 m/s (one can use a metronome and practice timing the movement of the rod) back and forth above the feeder over a horizontal distance of 50 cm, maintaining the hornet’s distance from the feeder at 2 cm, and tally the number of bee forager visits every 10 min.
  g. Execute six replicate tests for each colony, conducting only one test daily per colony within the time frame of 14:00–17:00.
  h. Carry out all experiments under sunny conditions, if possible, to ensure optimal bee foraging activity and waggle dancing.
    ***Note:*** Hornet foragers can also be captured from their nest entrance as they return or depart with insect nets but be very careful of being stung when working at hornet nest entrances.
    **CRITICAL:** Be very careful when handling hornets. Wear a protective hornet suit and gloves. Hornet venom can necrotize human tissues.
6. To observe and record the collective response of a colony to simulated hornet attacks, follow these steps:
  a. Begin by training bees to a feeder for 30 min, then proceed to document the colony’s response to hornet attacks at this feeder.
  b. Before introducing hornets near the feeder, count the total number of foragers, waggle dancers, and stop signals over 10 min to establish a baseline.
  c. Use a microphone, such as the Movo LV1 Lavalier Microphone, attached to a thin metal rod coupled with a camera, for example, a Sony HDR-PJ790, to record both waggle dances and stop signals.
    ***Note:*** The camera alone is sufficient to record waggle dances, but the stop signal can only be reliably detected by its sound and its visual behavior (see below).
    ***Note:*** Any small electret condenser microphone can be used.
  d. We recommend a video camera that can film at 25–30 frames per second and has a working distance of ≥50 cm (which can be reduced using inexpensive macro 2×–4× filter adapters).
    ***Note:*** Manual light gain function is desirable. However, the best measure of light sensitivity is the size of the CCD chip and the size of the lens. A larger lens allows more light to shine on the CCD and a larger CCD chip will be more sensitive. A minimum of a 2.5 cm CCD sensor is recommended.
  e. Situate the microphone and camera in proximity to the dance floor within the observation colony, as depicted in Figure 1 and detailed in Methods video S2**.**
    Methods video S2. How to detect a stop signal, related to step 6 In this video, you can see both the stop signal and the typical honey bee waggle dance in which the bee painted with a blue mark on its dorsal thorax is performing a classic figure-8 waggle dance for a feeder located 120 m from its colony. Carefully observe the bee that is painted purple/pink on its dorsal thorax. This bee was attacked by a hornet at a feeder while it fed on pure sucrose solution without any dopamine hydrochloride added: it delivers a stop signal to the waggle dancer. Note that a stop signal should be confirmed based upon two criteria, visual and acoustic: (1) the stop signaler pushes her head against the signal receiver and the receiver is visibly displaced or freezes in response to the signal, and simultaneouisly (2) the stop signaler produces the characteristic stop signal at the correct frequency and for the correct duration.^6^
  f. Gradually scan the microphone across the waggle dance floor, typically the comb closest to the nest entrance, maintaining about 1 cm distance from the bees.
  g. Navigate the microphone in a zigzag motion, moving up and down at roughly 0.2 m/s across the dance floor’s surface.
  h. Concurrently, have the person monitoring the hive record the total number of waggle circuits and stop signals every 10 min, utilizing the microphone and a Sony™ HDR-PJ790 camera focused on the dance floor, where the majority of waggle dances and stop signals are observed (refer to Methods video S2).
  i. If possible, rely on natural light from a window, or use an artificial light source when natural lighting is inadequate.
    ***Note:*** Red light could be used, but would typically need to be quite bright for most video cameras to detect sufficient detail. Low-intensity white light, either natural or artificial does not disturb bee behavior.^5^
  j. Real-time counting of stop signals and waggle dance circuits is feasible by connecting headphones to the video camera.
    ***Note:*** For enhanced accuracy, it is advisable to review the recordings, ideally by a trained observer who is not aware of the experimental conditions (blind data analysis), to tally the dance circuits and stop signals from the video footage.
    **CRITICAL:** A stop signal should be confirmed based upon two criteria, visual and acoustic: (1) the stop signaler pushes her head against the signal receiver and the receiver is visibly displaced or freezes in response to the signal, and simultaneously (2) the stop signaler produces the characteristic stop signal at the correct frequency and for the correct duration^6^ (Methods video S2**)**.
    ***Note:*** We are often asked if this experiment could be conducted with a honey bee observation hive enclosed in glass or even thin plastic.
    ***Note:*** It is not possible to determine the producers of stop signals accurately or detect them consistently with such an enclosure.
    ***Note:*** One can detect stop signals with vibrometers implanted in the nest, but determining the senders of these stop signals is quite difficult.
    ***Note:*** As noted below, a stop signal determination is based on two criteria, visual and acoustic.
    ***Note:*** Bees use multiple types of vibrational signals and making a correct and accurate determination is important.^7^
    ***Note:*** It may be possible to use fine black mesh such as tulle as a screen between the experimenter and the bees.
    We prefer not to do this because it visually obscures what can be seen and the scanning microphone can hit the tulle and disturb the bees if the scanner is not careful.
7. To accurately assess the colony-level forager responses both before and after simulated hornet attacks, adhere to the following protocol:
  a. During the control phase, before any attacks, pass a clean rod, without a hornet, at a distance of 2 cm from the marked foragers at the feeder.
    ***Note:*** In our experiments, we found no differences in the behavior of foragers who experienced this control clean rod as compared with foragers who experienced no rod and no hornet.
  b. Upon the targeted forager’s departure from the feeder, the person at the feeder should notify the observer at the hive through cell phones or two-way radios.
  c. This communication is to ensure readiness for tracking the forager’s return to the hive, allowing for the documentation of its waggle dancing, stop signaling, and the duration of its visit within the hive.
  d. Continue to observe and document these specific behaviors for the same individual forager following an encounter with a simulated hornet attack, as previously detailed (see Methods video S1).
  e. This process should be repeated for 10 bees from each of the three colonies under study.
    ***Note:*** Foragers exhibit a peak in stop signal production within the first 10 minutes following their return to the hive after a simulated attack, with the rate of stop signaling subsequently diminishing.^6^^,^^8^
  f. This observation period should, therefore, be based on the natural decline in the rate of stop signaling by individual foragers.
  g. During these focused observations, you should also evaluate the impact of stop signals on waggle dancers that receive these signals.
    ***Note:*** This involves counting the number of waggle dance circuits a forager performs during a visit to the hive, with the duration of a visit defined from the moment a forager re-enters the hive until its departure from the hive.
8. Here, we give instructions on how to measure individual forager responses before and after simulated hornet attacks.
  a. When a marked forager departs the feeder, the person monitoring the feeder uses a two-way radio or cell phone to inform the hive observer, ensuring they are prepared to document the forager’s waggle dancing, stop signaling, and the duration of its visit inside the hive upon its return.
  b. This procedure is then repeated after the forager has experienced a simulated hornet attack, to record these behaviors for 10 bees from each of the three colonies involved.
  c. It is important to remember that a forager produces the most stop signals immediately upon returning to the hive after an attack, with the frequency of these signals naturally decreasing thereafter.
  d. The observation period, typically lasting 10 min, should be based on this decline.
  e. Additionally, during these observations, the effect of stop signals on waggle dancers that receive these signals is recorded by counting the number of waggle dance circuits performed during a hive visit, which is defined as the period from when a forager returns to the hive until it leaves again.
  f. This comprehensive approach allows for a detailed analysis of how simulated predator threats influence forager behavior and communication within the colony.

***Note:*** In this individual monitoring procedure, you will be following individual bees as they return from the nest (Methods video S1). You are not scanning the entire dance floor.

**CRITICAL:** While following the returned forager inside the nest, make sure that you do not disturb it by touching it with the tracking microphone.

### Head dopamine levels of foragers after different predation and signaling treatments

**Timing: 14 days (approximately 7 h per day)**

9. To quantify dopamine levels in the heads of bees, follow these steps.
  a. Using entomological forceps, capture bees performing four distinct types of bee behavior inside the hive: control bees, waggle dancers, bees that emit stop signals, and bees that receive stop signals.
  b. Identify and document control bees as those that have frequented the feeder multiple times but have ceased visiting and remain within the hive for a period exceeding 1 h.
  c. Watch waggle dancers who persist in visiting the feeder and initiate their dance immediately upon entering the hive.
    i. The waggle dancer should be captured and frozen (see below) to measure its head dopamine levels after it has completed five waggle dance circuits.
    ii. Make sure that the waggle dancers that receive stop signals are performing waggle dances for the training feeder, but have not directly experienced hornet attacks or foragers that have sensed the presence of hornets at the feeder.
  d. Record the behavior of stop signalers, bees that continue to visit the feeder and encounter an attack by a tethered *V. mandarinia* hornet while collecting nectar, and start emitting stop signals upon their return to the hive.
  e. During the simulated hornet attacks, the individual monitoring the feeder is tasked with recording the identities of all bees at the feeder.
    ***Note:*** This is crucial to confirm that the waggle dancers, from which head dopamine levels are later measured, have not directly encountered the predator at the feeder.
  f. After the simulated attacks, it is necessary to substitute the feeder (both glass top and plastic base) with a fresh one containing the same volume of sugar solution (approximately 50 mL).
    ***Note:*** Conduct this step to eliminate any residual odors linked to predation, such as those from potential honey bee alarm pheromones or hornet scents, ensuring the integrity of subsequent observations.
  g. Capturing can be done by gently grabbing a bee with entomological forceps and immediately placing it in liquid nitrogen (see below).
    ***Note:*** Another possibility is to use an insect aspirator.
    ***Note:*** If you notice that other bees show disturbance when you capture the focal bee or smell honey bee alarm pheromone, you will need to be gentler in your technique.
    ***Note:*** It is possible to use a modified queen cage that opens like a clip to capture bees.
10. To process bee heads and measure dopamine (DA) levels, follow these steps.
  a. Immediately after capturing the bee, immerse it into liquid nitrogen to fix the head dopamine levels, then place it in a plastic 2 mL Eppendorf centrifuge vial and store it in a −80°C freezer until ready for processing.
  b. With a clean scalpel, carefully remove the antennas, eyes, mandibles, proboscis, and cuticles from each bee head.
  c. Introduce 500 μL of protein precipitation solution (composed of 0.4 mol/L HClO_4_, 2.6 mmol Na_2_S_2_O_5_, and 2.7 mmol EDTA) into a 1.5 mL microcentrifuge tube containing the remaining head tissue.
  d. Utilize an electric grinder (Tiangen) set at 8000 rpm to pulverize the head tissue for 1 min.
  e. Add 500 μL of the protein precipitation solution to the tube, then vortex and centrifuge at 4°C and 13,000 rpm (15,871 RCF, Eppendorf Centrifuge 5424R) for 30 min.
  f. Filter the supernatant through a 0.22 μm membrane, transferring the filtrate into 1.5 mL micro vials for subsequent analysis via High-Performance Liquid Chromatography with Electrochemical Detection (HPLC-ECD).
  g. The chromatographic system employed should consist of a solvent delivery pump (Waters 1525 binary HPLC pump), an autosampler (Waters 2707), and a C18 reversed-phase column (inner diameter 4.6 × 250 mm, with an average particle size of 5 μm) maintained at 39°C, coupled with a UV detector (Waters 2489, set to 264 nm).
  h. Record and analyze the signals from the detector with Breeze 2 software.
  i. Prepare the mobile phase with 100 mL of acetonitrile, 1.7 mmol of sodium 1-octane sulfonate, 64 mmol of anhydrous sodium dihydrogen phosphate, and 50 μmol EDTA, adjusting the pH to 3.0 with citric acid.
  j. Ensure the solution is free from impurities by filtering through a 0.22 μm pore diameter filter membrane and removing air bubbles with an ultrasonic instrument (SB-800D, Scientz, at 40 kHz ultrasonic frequency) for 30 min.
  k. Maintain a constant flow rate of 1.0 mL/min during detection.
  l. Employ dopamine hydrochloride standards to establish a baseline for quantifying the dopamine content in the bee head samples.
  m. Before each analytical run, inject a control sample along with three different concentrations of DA standards (0, 6, 60, and 600 ng/mL) to calibrate the system.
  n. Finally, determine the DA levels in each sample by comparing the peak areas of the sample with those of the standards.
  o. Measuring the amount of DA against a standard allows one to determine the absolute amount of DA.

***Note:*** It is crucial to measure the level of DA with respect to the amount of protein in the brain, a housekeeping protein, and total brain weight. These procedures allow researchers to better compare different bees since brains can be of different sizes.

***Note:*** One possibility is to use a Bradford protein assay^9^ to measure the protein concentration in a reserved fraction of the tissue used. One can then calculate the amount of dopamine per microgram of brain tissue.

**CRITICAL:** Ensure that your HPLC-ECD machine is correctly calibrated and in good working order as per manufacturer instructions.

### Effect of consuming dopamine on forager behaviors after hornet attacks

**Timing: 14 days (approximately 7 h per day)**

11. To test whether consuming dopamine in sugar solution and thereby elevating circulating dopamine levels would alter the behavior of bees after hornet attacks, follow these steps.
  a. Train individually paint-marked bees to approach feeders, applying the same methods used in steps 1–5.
  b. Collect bees from a single colony and randomly place them at two separate feeders.
    i. Position these feeders 120 m from the focal colony and ensure they are spaced 3 m apart from each other.
  c. During the training phase, fill both feeders with a 50% w/v sucrose solution to motivate the bees to return.
  d. Conduct two different types of trials. Conduct these trials on different days under similar weather conditions at the same feeder location, the location of the training feeder. Use pseudorandom alternation to ensure an equal number of control and experimental trials.
    i. For a control trial, replace the training feeder with a control feeder that continues to offer a pure 50% w/v sucrose solution with no other compounds.
    ii. For an experimental feeder, replace the training feeder with an experimental feeder that provides a 50% w/v sucrose solution with 100 μg/mL dopamine hydrochloride (Cas No. 62-31-7, sourced from J&K Scientific, China).
  e. This setup is designed based on the methodologies established by Harris and colleagues, aiming to assess the effects of dopamine supplementation on bee behavior and physiological responses.^10^ Dong et al.^1^ showed that such feeding would elevate the dopamine levels in bee heads from 6.9 ng/bee head to 8.2 ng/ bee head and that these levels could persist for over 1 min (the duration of time that bees took to return to the nest after feeding and show altered behaviors).
12. To study the effects of hornet attacks on bee behavior, follow these steps.
  a. Choose foragers that repeatedly visit the feeders over 30 min (at least 10 visits over this time period).
    ***Note:*** Capture foragers with an aspirator if they visit only infrequently.
  b. During one of these visits, randomly select foragers at the feeders and subject them to a simulated attack by a tethered *V. mandarinia* hornet (as described in step 7), initiated after the bees have been consuming the sucrose solution for 30 s.
  c. It is crucial to observe and record for how long bees continue foraging at the feeder after being attacked.
  d. Once the attacked bees leave the feeder, use two-way radios or cell phones to communicate the departure of the attacked forager back to the hive to the observer monitoring hive activities.
    ***Note:*** This communication allows the colony monitor to prepare to observe the forager's behavior upon its return.
  e. Upon the forager’s return to the hive, meticulously document the number of waggle dance circuits it performs and the number of stop signals that it generates.
  f. Additionally, assess the total duration of its presence inside the hive.
  g. These measures are essential to understanding the impact of predation threats on foraging behavior and intracolony communication, providing insights into the adaptive mechanisms bees employ in response to external stresses.
13. To estimate the rate of dopamine consumed by foragers per feeder visit, follow these steps:
  a. Begin by training ten bees from each of three different colonies, totaling 30 bees, to visit a feeder containing a sucrose solution mixed with dopamine, as described above.
  b. After training, capture these bees in a 10 mL vial and apply a brief CO_2_ anesthesia to temporarily immobilize them.
    i. Insert a tube from the CO_2_ tank into the vial and gently expose the bees to a low gas rate (1–2 L per minute) until movement ceases, which typically takes 1–2 min. Stop providing CO_2_ once movement ceases.^11^
  c. This method is effective for handling bees without causing long-term harm.
  d. Once immobilized, weigh each bee using an analytical balance (model ES1205A, Shanghai, China, or a similar model) with an accuracy of 0.01 mg to determine their pre-feeding mass accurately.
  e. After weighing, allow the bees to recover from anesthesia and return to foraging at the experimental feeder.
    ***Note:*** Providing bees with 95% oxygen at a flow rate of 1 L/min can help speed up their anesthesia recovery.
  f. Observe their behavior over several visits until they display no hesitation in approaching and landing on the feeder.
    ***Note:*** Hesitation is characterized by bees circling the feeder more than two times before landing, whereas no hesitation involves bees approaching and landing immediately.
  g. On their subsequent visit to the feeder, permit the bees to ingest the DA-laced sucrose solution for 30 s.
  h. Capture and anesthetize the bees with CO_2_ a second time, then weigh them once more to ascertain their post-feeding mass.
  i. Determine the amount of DA consumed by calculating the change in body mass to calculate the amount of sucrose solution imbibed and thus the amount of dopamine consumed.
  j. This approach provides insights into how DA affects foraging behavior and potentially influences energy intake and foraging efficiency in bees.
14. To measure the amount of DA in the brains of foragers after feeding on the dopamine hydrochloride sucrose solution, follow these steps:
  a. From the bees that have regularly visited the feeder and fed on the dopamine hydrochloride solution for 30 min, randomly select a subset, ensuring you have three replicates per colony, which amounts to six bees per colony across three colonies.
  b. Half of these bees should have ingested the DA-enriched sucrose solution.
    ***Note:*** This selection process should occur after the bees have fed for 30 s on the solution, following around 30 minutes of total feeding across multiple visits.
  c. Use liquid nitrogen for the freezing process, as this method instantly halts metabolic processes, preserving the physiological state of the bees at the time of their last feeding.
  d. This approach is crucial for subsequent analyses that aim to quantify the direct effects of DA ingestion on the bees' physiological condition and potential alterations in their metabolic or neural functioning.
  e. Follow the procedures for DA quantification.

## Expected outcomes

Bees not exposed to hornet attacks at feeders are expected to return to their nest and perform waggle dances, signaling resource locations to their colony. In contrast, bees experiencing attacks are predicted to emit stop signals upon returning to the nest and refrain from waggle dancing. Such encounters should influence brain dopamine levels, with non-attacked bees showing a temporary increase and attacked bees a decrease in dopamine from 5.6 ng/bee head to 3.1 ng/bee head. Bees that consume a dopamine-supplemented sugar solution for 30 min should maintain elevated dopamine levels regardless of subsequent attacks. These dopamine-fed bees, even when attacked, should not exhibit a strong aversive response. Instead, they will likely continue feeding, visiting the feeder, and performing waggle dances. Conversely, bees not supplemented with dopamine and that are subsequently attacked should demonstrate a strong aversive response, abandoning the feeder and emitting stop signals upon their return to the nest.

## Quantification and statistical analysis

To process and interpret the data, use the following procedures. One can use JMP statistical software (SAS Institute) or similar software such as R. With any statistical software, it is essential to understand how the software is making the intended calculations and the limitations and assumptions inherent in these calculations and models. Consult a qualified statistician if there are any doubts. To assess colony-level data, apply Univariate Repeated Measures Models, considering the colony as the repeated measure and the experimental phase as a fixed effect. Log-transform the number of stop signals and feeder visits per 10 min, if needed, to meet the assumptions of normality and homoscedasticity. For analyzing individual behaviors such as the production of stop signals, waggle dance circuits, and duration of hive visits, implement Repeated Measures Mixed Models with the REML algorithm. This model will incorporate the colony as a random effect and individual bee identity nested within the colony also as a random effect, with the experimental phase as a fixed effect. Log-transform the number of stop signals, waggle dance circuits, and hive visit duration, if needed, based on the distribution of model residuals to ensure the appropriateness of your statistical models. To explore the impact of bee type on brain dopamine levels use a Mixed Model with the REML algorithm. The model will feature colony as a random effect and bee type as a fixed effect to effectively differentiate between control and experimental groups. To investigate the influence of dopamine supplementation, use another Mixed Model with the REML algorithm. This model considers the colony as a random effect and the treatment type (control sucrose solution versus sucrose solution with dopamine) as a fixed effect. As appropriate, based upon inspection of the model residuals, log-transform variables such as time on the feeder, time inside the hive, the number of stop signals produced, and the number of waggle circuits.

Finally, to assess the effect of dopamine supplementation on bee head dopamine level, use a Mixed Model with the REML algorithm. The model includes colony as a random effect and bee type (bees feeding on control sucrose solution versus those on the experimental sucrose solution with dopamine) as a fixed effect, allowing for a clear comparison between the groups.

## Limitations

Multiple honey bees studies have used HPLC-ECD to measure dopamine levels in bee brains.^12^^,^^13^ However, HPLC-MS is a more sensitive method for measuring dopamine levels. Nonetheless, HPLC-ECD has been successfully used and, given the large changes in dopamine levels detected by multiple studies, evidently suffices.^12^^,^^13^

We also suggest the following modifications: measure only brain tissue and not simply focus on head tissue from which the antennas, eyes, mandibles, and proboscis and cuticle have been removed.

## Troubleshooting

### Problem 1

It may be difficult to train bees at different times of year, depending upon the availability of natural food (before you begin step 12).

### Potential solution

A classic training technique, though slower, is to place the feeder in contact with the colony entrance and have bees walk onto the feeder as they leave the nest. Slowly increase the gap between the feeder and the nest entrance. At first, moving in 1 cm units, but once bees begin to fly to the feeder, by a few meters at a time. Make sure that all of your trained bees return to the feeder before you move it again. If bees do not return, move the feeder back to its last position, wait until they return, and then reduce the distance you move it. Keep track of all bees visiting the feeder on a census sheet.^5^

### Problem 2

Bees do not always waggle dance (before you begin step 12).

### Potential solution

Choosing a time of relative natural food dearth is helpful. Please consult local data on when the fewest floral resources are available. If you cannot observe consistent waggle dancing, then increase the sucrose concentration. In some cases, a saturated sucrose solution (2.5 M) can be used. If this still does not elicit waggle dances, you will need to conduct these experiments at a different time of year (step 12). Detailed maps of the quantity and quality of floral resources that are used by bees are not usually available for most areas. However, Dornhaus and Chittka provide useful spatial data in different geographic regions.^14^ As an example of seasonal floral dearth for the region around La Jolla, California, Park and Nieh^15^ provide detailed maps of where pollen foragers found pollen that they waggle danced for throughout the year. Such data is typically best obtained by watching the bees and seeing when and for what locations they waggle dance.^15^

### Problem 3

You have chosen a time of food dearth and other colonies of bees find your feeder and begin crowding out and fighting your trained bees for this resource (before you begin step 12).

### Potential solution

• This scenario, regrettably, is not uncommon. Once this has occurred, you typically need to aspirate and capture all of the bees on the feeder (since you do not know which unmarked bees are from your colony or other colonies), freeze them, and then begin training again to a new location to prevent this from happening again.
• Be extremely careful and stringent during training. Every bee that lands on the feeder must be uniquely marked and verified upon its return to the focal colony. If that bee is not observed in the focal colony, immediately capture it with an aspirator upon its next return to the feeder and freeze it at the end of the day. This procedure might sound extreme but, in fact, you are limiting the sacrifice of bees by reducing recruitment from non-focal colonies (step 3).
• Do not train bees to any location that you have previously used and that has been visited by non-focal bees (step 1).
• Reduce the sucrose concentration to the minimum necessary to limit recruitment (step 3). Recruitment increases for higher concentration sucrose solution. Thus, using the minimum concentration necessary for trained foragers to return to a feeder is helpful since it reduces the possibility of massive recruitment by non-focal colonies, and it also controls the number of foragers from focal colonies.
• Keep in mind that your observation colony only has two combs, whereas standard bee colonies contain 8–10 combs and feral colonies can be even larger. This means that a non-focal colony has a much larger workforce to overwhelm and take over your feeder.
• If possible, work in an area with few other honey bee colonies (step 12).

### Problem 4

Hornets elicit variable responses from bees (step 5).

### Potential solution

Hornets are the most alarming to honey bees when they move and buzz their wings, as in flight. Hornets that are not moving should be replaced. To keep them in good condition, we recommend freshly harnessing them, and if needed, feeding them with sugar solution through a syringe. Remember to be careful when handling hornets to avoid being stung.

### Problem 5

No bees return to the feeder after you present a hornet at the feeder (step 5).

### Potential solution

Depending upon natural food availability, bees may show aversion even to the presence of a hornet. If this occurs, you may wish to choose a different time to run these experiments, perhaps when natural food is even more scarce and bees are therefore willing to take more risks. Another possibility is to reduce the number of bees that experience the hornet at the feeder. For example, wait for only one bee to be present on the feeder when you present the hornet.

## Resource availability

### Lead contact

James Nieh (jnieh@ucsd.edu).

### Technical contact

Dong Shihao (dongshihao@xtbg.ac.cn).

### Materials availability

The materials used were purchased from standard chemical suppliers, who are listed in the materials and equipment table. No custom materials were created or used.

### Data and code availability

The data for the study on which this protocol is based is publicly available at Zenodo.com (https://doi.org/10.5281/zenodo.7758140).
